# The virome: a missing component of biological interaction networks in health and disease

**DOI:** 10.1186/s13073-016-0287-y

**Published:** 2016-04-01

**Authors:** Scott A. Handley

**Affiliations:** Department of Pathology and Immunology, Washington University School of Medicine, Saint Louis, MO 63110 USA

## Abstract

Host-associated viral populations, viromes, have been understudied relative to their contribution to human physiology. Viruses interact with host gene networks, influencing both health and disease. Analysis of host gene networks in the absence of virome analysis risks missing important network information.

## The virome interaction network

The human virome consists of viruses that infect eukaryotic cells (eukaryotic viruses) and bacteriophages. The gut virome is a viral collective inhabiting the intestine, co-existing and closely integrated to the bacterial microbiome, fungi and other microbial communities that constitute the microbiome. In addition, due to the integrative capacity of many viruses, host genomes are frequently filled with virus-derived genetic elements (retroviral elements in eukaryotic genomes and prophages in bacterial genomes). Viruses can be found on all mucosal surfaces, and frequently persist in other cell types, such as is seen with chronic herpesvirus infection of neuronal cells. It is estimated that, in addition to integrated chromosomal viruses, each individual healthy human harbors more than ten permanent chronic eukaryotic viral infections that drive continuous activation of the immune system (for a full review of virome influences on mammalian physiology see [1]). These most commonly include herpesviruses, polyomaviruses, anelloviruses, adenoviruses, papillomaviruses and, for many people, additional viruses such as hepatitis B virus, hepatitis C virus, and HIV. However, metagenomic analysis of virus-like particle preparations from human samples suggests that the number and types of human-associated viruses is underestimated. Virus-like particle metagenomic sequencing has repeatedly shown that only 14–87 % of sequences can be classified, suggesting that a great number of uncharacterized viruses reside within and on us [2]. Continued analysis of metagenomic data sets using novel approaches to characterize this metagenomic “dark matter” will likely uncover more novel viruses, as exemplified by the discovery of a highly abundant bacteriophage (crAssphage) ubiquitous to human fecal metagenomes [3].

## The virome as a missing component of biological networks

Systems biology provides a way to derive a holistic view of the role of the microbiome in health and disease. Such work relies on mapping the interaction between complex biological systems such as the bacterial microbiome network with other, less studied, components of the microbiome such as the virome.

**Fig. 1:**
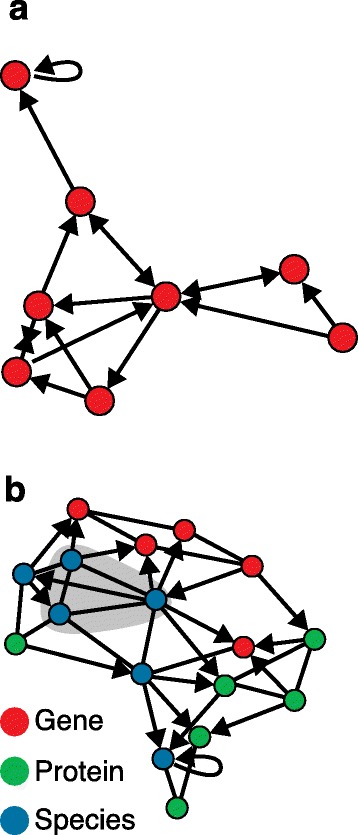
Examples of biological interaction networks. **a** Single data type network (for example, genes). Nodes are represented by circles, and edges (interactions) as *black arrows*. **b** Mixed-type biological network showing interactions between genes, proteins and species (for example, viruses, bacteria). Masked area (*gray*) indicates missing subgraph (for example, viral taxa)

These networks can be delineated using high-throughput and reductionist experimentation to define the nodes and edges (Fig. 1).

A deeper understanding of how the microbiome impacts human function requires an understanding of the function of the human virome network. It is interesting to note that, of the US$920 million invested in microbiome research from 2012 to 2014, only 3 % of research was dedicated to studies of viromes [4]. Extrapolating available viral diversity data leads to an estimate that there are approximately 320,000 mammalian viruses awaiting discovery [5]. This number is completely overshadowed by the estimates for the numbers of viruses that infect bacterial cells (bacteriophages), with estimates as high as 10^31^ bacteriophages on the planet, outnumbering their bacterial hosts at a 10:1 ratio [6]. In comparison to studies of the bacterial microbiome, the relative paucity of studies on viral diversity undermines our capacity to understand full biological networks.

Metagenomic analysis will continue to classify an extensive menagerie of both eukaryotic viruses and bacteriophages. This information will populate the nodes of the biological interaction network, but will be largely valueless for characterizing graph interactions. The generation of interaction networks will require classical experimental approaches, including mouse and tissue culture infection models for characterizing eukaryotic viruses, and bacterial host infection studies for bacteriophages. Both of these strategies are extremely challenging and rely on the isolation of pure virus and the availability of a susceptible model host.

The selection of a model host organism to study eukaryotic virus–host interactions is largely determined by the availability of laboratory models, with mouse being the most prevalent. The lack of replication in mice would severely hamper the study of virus–host interactions. Bacteriophage–host determination is equally challenging. Computational predictions of bacteriophage hosts are largely unsuccessful, with the capacity to assign 1 bacteriophage to 1–4 possible bacterial hosts in only 10–40 % of cases [7]. While computational methods will certainly mature, mechanistic studies also require the growth of individual bacteriophages in susceptible bacterial hosts. While challenging, these mechanistic studies will incrementally populate certain portions of the overall interaction network (virus–gene, bacteriophage–virus interactions).

## Virome interactions in health and disease

As the virome interaction network begins to be integrated with other biological interaction networks, we will be able to assess more complex biological scenarios. An example of this would be transkingdom interaction networks in which organisms from different domains of life influence one another through networks of interactions. For instance, helminth infection has been shown to alter the host immune response, favoring herpesvirus reactivation, providing insight into the helminth mammal–virus interaction network [8]. Recent work has also shed light on the expansion of gastrointestinal bacteriophage populations, which inversely correlates with changes in bacterial diversity during inflammatory bowel disease (virus–bacteria–mammal interaction network) [2]. This work was important in showing a contributory role of the enteric virome in inflammatory bowel disease and bacterial dysbiosis, providing evidence that the virome must be considered in studies of the bacterial microbiome. As such, longitudinal studies to assess the dynamics between the virome and bacterial microbiome in early life have been performed [9, 10], and we are now beginning to uncover this relationship and how it may be modulated. Together, these works have major implications for how we approach microbiome diagnostics, as the virome is intimately connected to the bacterial microbiome, and focusing on one without investigating the impact of the other could lead to an incomplete and even misinformed understanding of healthy and diseased states.

We therefore conclude that high-throughput characterization of viromes combined with mechanistic studies integrating virus interactions with host biological networks will enhance our ability to assess human biology for what it truly is: an ecosystem of biological networks contributing to overall health when acting in concert or disease when disrupted.
